# Integrating grafting and bio-inputs for sustainable management of root knot nematode, *Meloidogyne incognita*, in tomato cultivation

**DOI:** 10.3389/fpls.2025.1623444

**Published:** 2025-08-29

**Authors:** Seenivasan Nagachandrabose, Mookiah Shanthi, Sankaran Pagalahalli Shanmugam, Thiyagarajan Elaiyabharathi, Radhakrishnan Sharmila, Kandasamy Devrajan, Ravishankar Manickam, Ramasamy Srinivasan

**Affiliations:** ^1^ Department of Nematology, Tamil Nadu Agricultural University, Coimbatore, India; ^2^ Centre for Plant Protection Studies, Tamil Nadu Agricultural University, Coimbatore, India; ^3^ Department of Agricultural Entomology, Tamil Nadu Agricultural University, Coimbatore, India; ^4^ World Vegetable Center South and Central Asia, Hyderabad, Telangana, India; ^5^ World Vegetable Center, Shanhua, Tainan City, Taiwan

**Keywords:** tomato grafts, root-knot nematode, host plant resistance, biorational inputs, integrated nematode management

## Abstract

**Introduction:**

Root-knot disease in tomato, caused by *Meloidogyne incognita*, presents a major challenge to global tomato production. This study explored a sustainable management approach by evaluating host-plant resistance through grafting combined with bio-inputs in farmers’ fields with high natural infestations of *M. incognita*.

**Methods:**

The commercial F1 hybrid Shivam® tomato was grafted onto bacterial wilt-resistant eggplant rootstocks, EG 203 and TS 03. Two field experiments were conducted with six treatment groups to compare the performance of 'EG 203-tomato' and 'TS 03-tomato' grafts against the non-grafted hybrid tomato, both with and without bio-input applications. The bio-input protocol included soil application of neem cake (250 kg/ha) and soil and seedling drenching at nursery and transplant stages using biocontrol agents (Bacillus subtilis, *Trichoderma asperellum*, and *Purpureocillium lilacinum*, each at 5 g/L).

**Results:**

Results indicated that the 'EG 203-tomato' graft demonstrated strong resistance to M. incognita, while the 'TS 03-tomato' graft remained susceptible, akin to the non-grafted Shivam® hybrid. The EG 203-tomato graft treated with the bio-inputs achieved the highest suppression of *M. incognita*, with reductions of 76.8–77.7% juvenile populations in the soil, 62.0–66.1% in female populations within roots, 73.6–77.3% in egg masses per female, and 38.1–40.0% in eggs per egg mass. This treatment also resulted in the lowest root gall index, measured at 2.0–2.1.

**Discussion:**

In both trial locations, 'EG 203-tomato' graft plants enriched with bio-inputs outperformed the non-grafted tomato in growth and yield metrics, achieving greater plant height (54.6–54.7 cm), leaf count (81.3–84.3 per plant), branch count (3.1–3.7) and fruit yield (10.8–11.5 kg/plant). These findings support the recommendation of EG 203-tomato grafts with bio-input management as an effective large-scale strategy for tomato growers combating *M. incognita* infestations.

## 1 Introduction

Tomato (*Solanum lycopersicum* Miller), from the Solanaceae family, is the world’s most cultivated vegetable crop. Its fruits are highly valued both fresh and processed, as they provide essential vitamins and minerals, including vitamin E (alpha-tocopherol), vitamin C, vitamin A, potassium, magnesium, and folate. Additionally, tomatoes contain phytochemicals like lycopene and β-carotene, which have been linked to potential health benefits, including reduced risks of prostate cancer and cardiovascular diseases (Gatahi, 2020). Tomatoes cultivation spans approximately 168 countries, with a global production reaching 186.82 million tonnes across 5 million hectares (FAOSTAT, 2024). China leads production, followed by India, Turkey, the United States, Egypt, and Italy. India alone contributes approximately 20.33 million tonnes annually, covering 0.841 million ha (Ghalawat et al., 2024). Major tomato-growing regions in India include Tamil Nadu, Andhra Pradesh, Madhya Pradesh, Karnataka, Gujarat, Bihar, Odisha, West Bengal, Telangana, Chhattisgarh, Haryana, Uttar Pradesh, and Maharashtra (Tiwari et al., 2022).

Despite its economic value, tomato productivity is hindered by various pests and diseases. Among the major constraints, the root-knot nematode *Meloidogyne incognita* (Kofoid & White, 1919) is considered one of the most destructive pathogens affecting tomato cultivation, owing to its global prevalence, highly specialized parasitic behavior, and persistent yield suppression. After hatching from soil-borne eggs, second-stage juveniles (J2) of *M. incognita* invade feeder roots and puncture plant cells using their stylet. Upon reaching the vascular tissues, they establish a sedentary lifestyle and induce the formation of multinucleated giant cells in the root endodermis to facilitate continuous nutrient uptake. They reproduce parthenogenetically, forming egg masses containing 200–400 eggs on the root surface within 25–30 days of infection.This parasitic activity disrupts the uptake of water and nutrients, leading to nutrient imbalances that cause stunted growth and significant yield losses.Globally, *M. incognita* can reduce yields by 10-85% in tomato, with Indian losses ranging between 40-91% (Nagachandrabose and Baidoo, 2018). Additionally, *M. incognita* predisposes plants to pathogens like *Fusarium oxysporum* and *Ralstonia solanacearum*, forming a destructive nematode-disease complex (Parrado and Quintanilla, 2024).

Managing *M.incognita* remains a significant challenge due to its soil- and root-dwelling nature, sophisticated parasitic mechanisms, prolific reproduction, and broad host range. Although chemical nematicides such as DD mixture, DBCP, and carbofuran have historically been employed, growing concerns over environmental safety, human health risks, and stricter regulations have prompted a shift toward eco-friendly alternatives. As a result, sustainable, non-chemical strategies such as the use of nematode-free planting material, resistant cultivars, organic amendments, biological control agents, and heat-based treatments are gaining importance in tomato nematode management (Rawal, 2020). However, individual eco-friendly approaches typically result in only 10–40% suppression of nematode populations, which is often inadequate under high-infestation conditions (Seenivasan, 2017). Therefore, an integrated approach combining multiple cost-effective, practical, and high-efficacy methods is essential for sustainable management of *M. incognita* in nematode-endemic tomato-growing regions.

Host plant resistance provides an economical and environmentally safe approach to manage phytonematodes in crops. Tomato resistance to *M. incognita* has focused on the *Mi-1* gene from *Solanum peruvianum*, which confers resistance to *M. incognita* (Wubie and Temesgen, 2019). However, Mi-1 resistance is temperature-sensitive and loses efficacy above 28°C, with some *M. incognita* populations overcoming this resistance, highlighting the need for alternative breeding approaches. The use of resistant rootstocks grafted onto commercial tomato scions has emerged as a promising, sustainable alternative to conventional and transgenic breeding (Shanmugam et al., 2024). Frey et al. (2020) demonstrated successful grafting of tomato using ‘Garden Gem’ as the scion and ‘Multifort’ as the rootstock, resulting in resistance to *M. incognita* under high temperatures and resistance to Fusarium wilt, enhancing growth and fruit yield. Grafting tomato scions onto the resistant rootstock *Solanum sisymbriifolium* (wild brinjal) has also effectively controlled *M. incognita*, significantly increasing fruit yields compared to non-grafted tomato plants (Baidya et al., 2017).

Neem (*Azadirachta indica* L.) is widely recognized for its pesticide properties, and neem cake, a byproduct of neem seed oil extraction, has demonstrated notable nematicidal effects. Soil amendment with neem cake significantly reduces *M. incognita* populations and promotes tomato growth (Meena et al., 2021). Among the eco-friendly strategies, biological control holds strong potential for sustainable nematode management. Notable biocontrol agents include *Bacillus subtilis* (Ehrenberg 1835) Cohn 1872, a plant growth-promoting rhizobacterium (Sohrabi et al., 2020); *Trichoderma asperellum* Samuels, Lieckfeldt & Nirenberg 1999, an antagonistic fungus (Sohrabi et al., 2020); and *Purpureocillium lilacinum* Thom., a nematode egg-parasitic fungus (El-Ashry et al., 2021). While these agents have demonstrated effectiveness under laboratory and greenhouse conditions, their performance often declines under field conditions due to competition with native soil microflora. Therefore, to enhance their persistence and efficacy in field applications, the incorporation of organic amendments is recommended.

This study aimed to evaluate the response of tomato grafted onto *Solanum melongena* (bacterial wilt-resistant eggplant) rootstocks to *M. incognita* populations in field conditions. Additionally, the effectiveness of grafting in combination with promising bio-inputs, including neem cake, *B. subtilis*, *T. asperellum*, and *P. lilacinum*, was assessed as an integrated management strategy for *M. incognita* in heavily infested tomato fields.

## 2 Materials and methods

### 2.1 Experimental site

Two field experiments were conducted from October 2022 to March 2023 in Coimbatore, Tamil Nadu, India. The first experiment was carried out in a farmer’s field at Vandikaranur village (11.006123° N, 76.830208° E) at an elevation of 435 m above mean sea level. The site featured red loamy soil with a pH of 7.13, organic matter content of 0.85%, electrical conductivity of 0.45 dS/m, and nutrient levels of 259 kg/ha nitrogen (N), 46 kg/ha phosphorus (P), and 569 kg/ha potassium (K). The experimental area spanned 1860 m^2^.

**Figure 1:**
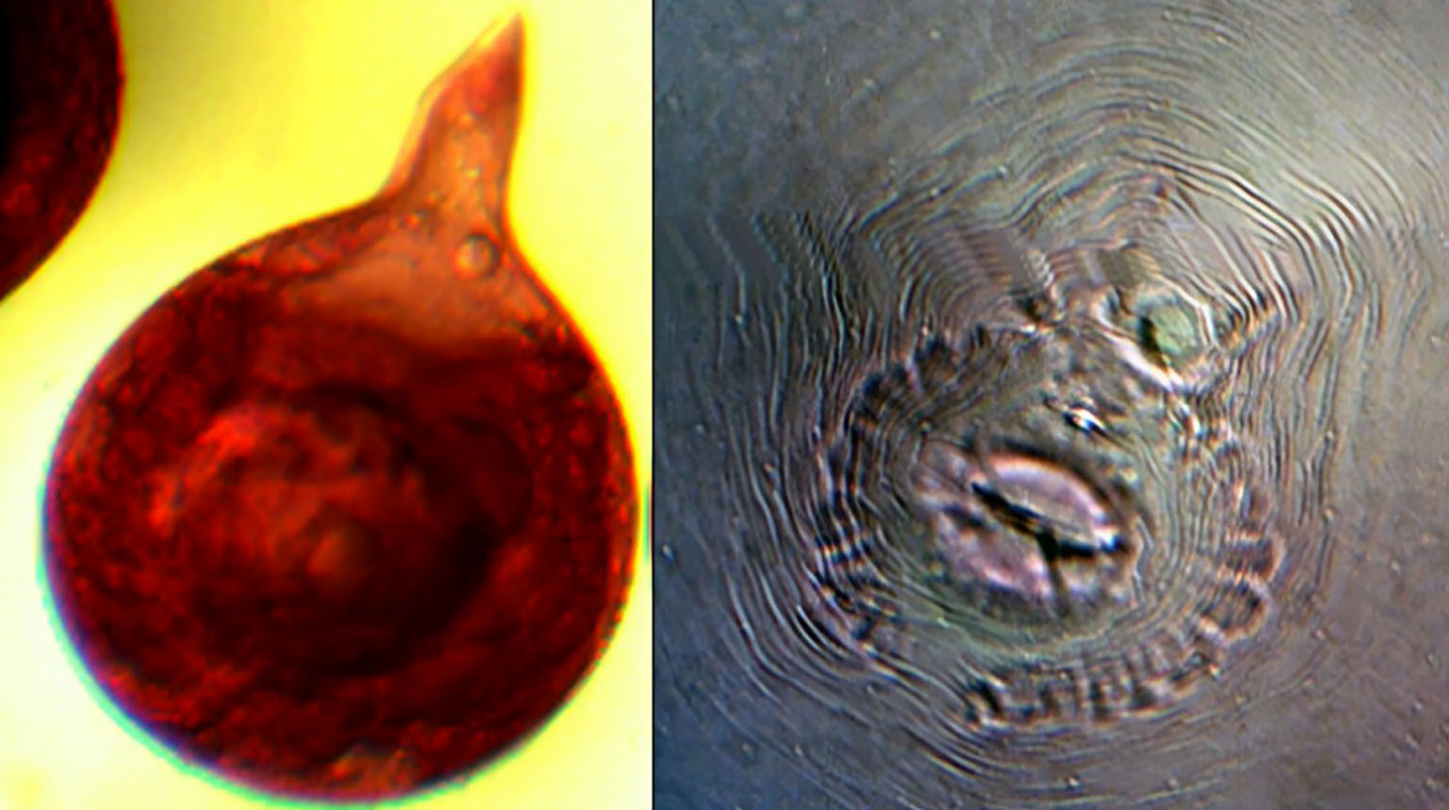
Left: *Meloidogyne incognita* adult female, Right: Perineal cuts of *M. incognita* female.

The second experiment was conducted concurrently in a farmer’s field at Karadimadai village (10.929349° N, 76.854019° E), located 431 m above mean sea level. The red loamy soil had a pH of 7.04, an organic matter content of 0.79%, an electrical conductivity of 0.41 dS/m, and nutrient levels of 242 kg/ha N, 41 kg/ha P, and 527 kg/ha K. The experimental area covered 1274 m^2^. Both sites had a history of continuous tomato cultivation and were naturally infested with *Meloidogyne incognita*. To confirm species identity, pear-shaped adult females were dissected from tomato roots, and their perineal patterns were examined under a compound microscope (40×) after mounting in anhydrous glycerin. All tested specimens displayed diagnostic features of *M. incognita*, including a high dorsal arch, coarse zigzag striae, and a characteristic tail whorl (Hartman and Sasser, 1985) (**Figure 1**).

### 2.2 Plant materials

Seeds of bacterial wilt-resistant eggplant (*Solanum melongena*) genotypes, specifically EG 203 and TS 03, were sourced from the World Vegetable Center, South and Central Asia, at the ICRISAT Campus in Hyderabad, Telangana. These genotypes served as rootstocks for grafting with the commercial tomato F1 hybrid Shivam^®^ (HyVeg, Coimbatore, India). The Shivam^®^ F1 hybrid features a determinate to semi-determinate tall growth habit, flat-round fruits with a green shoulder, and a weight of approximately 90–100 g. The fruits are firm, acidic in taste, and reach maturity 62–67 days after transplanting. The hybrid exhibits moderate resistance to the tomato leaf curl virus (ToLCV).

### 2.3 Bio-inputs

The neem cake utilized in this study was procured from the Kovai Oil Expeller Unit in Coimbatore, India. It contained Azadirachtin (850 ppm) along with macronutrients, including Nitrogen (1.5–3.0%), Phosphorus (0.5–1.55%), and Potassium (0.5–1.0%). Talc-based commercial formulations of *Bacillus subtilis* Bbv57 (2.5 x 10^8^ cfu/g) and *Trichoderma asperellum* Tv1 (2.8 x 10^6^ cfu/g) were sourced from the Department of Plant Pathology, Tamil Nadu Agricultural University, Coimbatore, India. Similarly, the talc-based formulation of *Purpureocillium lilacinum* TNAUPL1 (2.8 x 10^6^ cfu/g) was obtained from the Department of Nematology at Tamil Nadu Agricultural University, Coimbatore, India.

### 2.4 Treatment components

Both field experiments included six treatment groups: (1) eggplant EG 203-tomato Shivam^®^ graft with bio-inputs, (2) eggplant TS 03-tomato Shivam^®^ graft with bio-inputs, (3) tomato Shivam^®^ with bio-inputs, (4) eggplant EG 203=tomato Shivam^®^ graft without bio-inputs, (5) eggplant TS 03–tomato Shivam^®^ graft without bio-inputs, and (6) tomato Shivam^®^ alone (non-treated control). In treatments involving bio-inputs, the protocol consisted of drenching the nursery growing medium with *B. subtilis*, *T. asperellum*, and *P. lilacinum* at 5 g/L, applied seven days after grafting. Subsequently, at the time of transplanting, seedlings received the same bio-input treatment, followed by a 30-minute shade-drying period prior to planting.

### 2.5 Seedling preparation and grafting process in nursery

In both experiments, tomato seedlings and eggplant rootstock were raised at a farmer-owned commercial nursery in Thondamuthur village (10.9899° N, 76.8409° E), Coimbatore, Tamil Nadu. Eggplant rootstock seeds were sown in 98-cell seedling trays sourced from Ms. Kaveri Agri Products, Krishnagiri, India, in July 2022, using decomposed coir pith supplied by M/s. RAR Coir Industries, Salem, Tamil Nadu, is the rooting medium. After sowing, the trays were stacked and covered with polythene sheets to maximize germination. Polythene was removed after three days, and the trays were arranged inside a shade-net house maintained at 30 ± 2°C, relative humidity of 60 ± 5%, with a 12.5:11.5 h light-to-dark cycle. Trays were irrigated thrice daily using a 500–750 mL sprinkling can.

Tomato Shivam^®^ seeds were sown seven days later, following uniform nursery practices for both crops. For bio-input treatments, seedlings were drenched with a mixture of *B. subtilis*, *T. asperellum*, and *P. lilacinum* (5 g/L each) using an atomizer to saturate the seedling beds. This drenching was conducted at 16 days after sowing (DAS) for eggplant and 14 DAS for tomato. Non-bio-input treatments received plain water. No pest or disease incidence occurred during nursery growth.

Healthy 30-day-old eggplant seedlings (rootstock) and 21-day-old tomato seedlings (scion) with a 1.5–1.8 mm stem diameter were selected for grafting. Both scion and rootstock stems were cut at a 30° angle just above the cotyledon level to create slanted, matching surfaces with uniform stem thickness. The cut surfaces were then aligned and secured together using grafting clips(Ms. Varsha Enterprises, Bengaluru, India), following the method of Black et al. (2003). Grafted seedlings were kept in shade-net healing chambers for 8–10 days at 90% relative humidity to facilitate graft union and then transferred to a standard shade-net house for three days to harden. Once hardened, they were ready for field transplantation.

### 2.6 Field setup and planting

Both experimental fields were ploughed twice to achieve a fine tilth. The trials were conducted using a completely randomized block design (CRBD) with four replications, and each plot measured 10 m x 5 m, with a 3 m buffer zone. In bio-input plots, neem cake was incorporated into the topsoil at a rate of 250 kg/ha before planting. Farmyard manure (12.5 t/ha) and single superphosphate (1172 kg/ha) were applied uniformly across all plots before planting. Nitrogen (200 kg/ha) and potassium (250 kg/ha) were used in 5–6 split topdressings in 5-6 divided doses.

Grafted eggplant-tomato and tomato seedlings were carefully transported to the fields. A solution of 20 L water containing *B. subtilis, T. asperellum*, and *P. lilacinum* at 5 g/L each was prepared for seedling drenching treatments. Bio-input-treated seedlings were soaked in this solution and dried in the shade for 45 minutes.

Raised beds measuring 90 cm in width, with a 30 cm spacing between beds, were prepared in the plots. Drip irrigation lines were installed along each bed, and seedlings were planted at 120 × 60 cm spacing, with 70 plants per plot in both experiments. The agronomic practices were adhered to the Tamil Nadu Agricultural University Crop Production Guide 2022 (Anonymous, 2022).

Low to moderate infestations of pests and diseases were observed, including leafhopper (*Amrasca biguttula*), thrips (*Thrips tabaci*), whitefly (*Bemisia tabaci*), *Phthorimaea absoluta*, *Liriomyza trifolii*, *Spodoptera litura*, *Helicoverpa armigera*, bacterial leaf spot, early blight, Fusarium wilt, tomato leaf curl virus, and tomato mosaic virus. Pesticides were applied as needed following the crop production guide recommendations.

### 2.7 Soil sampling and nematode density assessment

The population density of *M. incognita* was assessed in each plot before planting and at harvest. Composite soil samples weighing 2.5-3.0 kg were collected per plot using a core sampler with a 1.0 cm diameter and 15 cm length. Twenty-five to thirty core samples were taken from each plot to form a composite sample. The composite samples were thoroughly mixed, and a 200 cm³ subsample was extracted by Cobb’s sieving technique, followed by the modified Baermann funnel method (Southey, 1986). Nematode populations were quantified by examining the suspension under a stereo zoom microscope (Kozo Zoom 645) at 40x magnification (Nagachandrabose et al., 2024).

### 2.8 Nematode infection assessment in roots

Five plants per plot were randomly selected following a zig-zag pattern and carefully uprooted at harvest. Roots were gently washed to remove adhering soil particles, and the root gall index was evaluated using a 1-5 scale (Taylor and Sasser, 1978): 1 = no galls/plant; 2 = 1–10 galls/plant; 3 = 11–30 galls/plant; 4 = 31–100 galls/plant; 5 = more than 100 galls/plant.

Secondary roots from each selected plant were collected, cut into 1 cm segments, and mixed thoroughly. A 1-g subsample was used to assess *M. incognita* female populations, egg mass count per gram of root, eggs per egg mass, egg mass parasitization, and biocontrol agent colonization.

To assess female *M. incognita* counts, 1-g root subsamples were stained with acid fuchsin-lactophenol and destained with plain lactophenol. The stained females were counted under a stereo-zoom microscope (40x magnification). For egg mass counts, 1-g subsamples were placed in a 90 mm Petri dish with water and examined directly under the microscope. To determine eggs per egg mass, 10 egg masses per treatment were dissected, placed in distilled water, crushed with a needle, and counted under the microscope at 40x magnification.

### 2.9 Re-isolation of introduced bioagents

**Figure 2:**
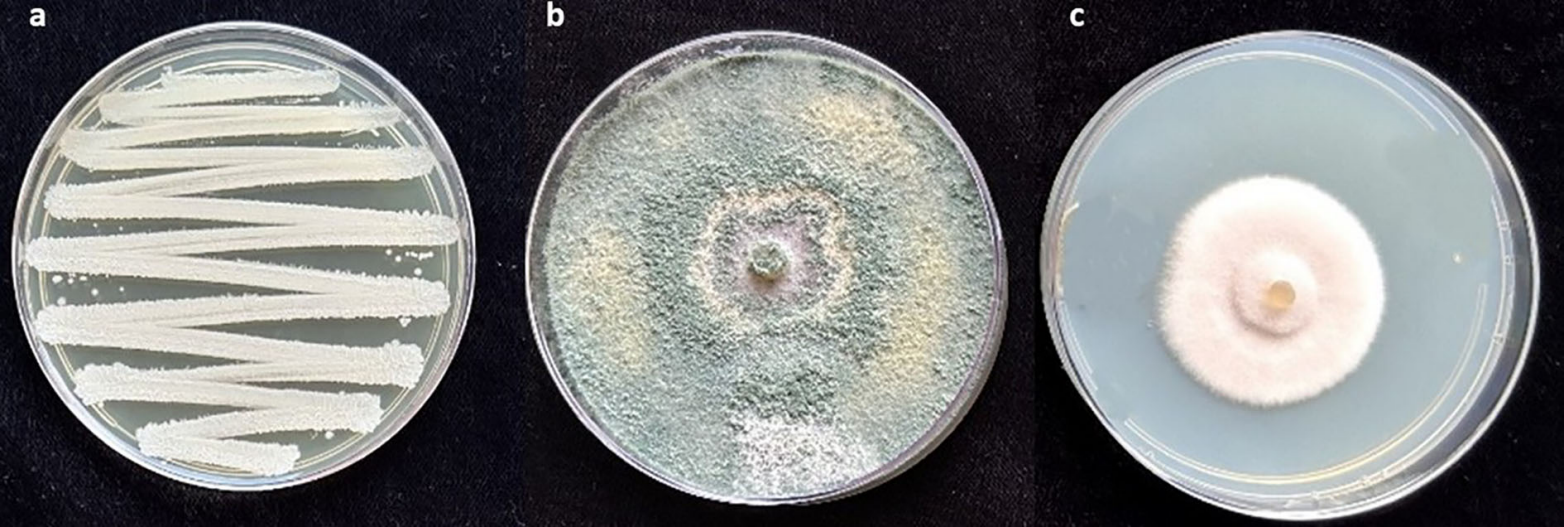
**(a)** *Bacillus subtilis* colonies showing round or irregular shapes, thick, opaque, and cream-coloured; **(b)** *Trichoderma asperellum* colonies with green, woolly, or cottony appearance; **(c)** Colonies of *P. lilacinum* with lilac-to-purple pigmentation and velvety texture.

To evaluate root colonization of *B. subtilis*, 1-g root subsamples from each treatment plot were surface-sterilized with 1% NaOCl and rinsed twice with distilled water. The sterilized roots were ground using a sterile pestle and mortar with 1 mL of distilled water, and the resulting slurry was transferred to test tubes containing 9 mL of distilled water. Serial dilutions were prepared up to 10^7^, and the *B. subtilis* was isolated by plating the dilutions onto nutrient agar media. The plates were incubated at 28 ± 3°C for three days, after which colonies were examined. *B. subtilis* colonies were identified based on their distinct morphology, round or irregular shapes, thick, opaque, and cream-coloured, and were quantified as CFU/g root (Nagachandrabose et al., 2022) (**Figure 2a**).

Subsamples of *P. lilacinum* and *T. asperellum* were cultured on potato dextrose agar (PDA) and yeast molasses agar (YMA), respectively. Root sections were incubated at 28 ± 3°C for 15 days. Colonies of *P. lilacinum* were identified by their lilac-to-purple pigmentation and velvety texture (**Figure 2c**), while *T. asperellum* colonies were recognized by their green, woolly, or cottony appearance (**Figure 2b**). The bio-agents were quantified in terms of colony-forming units (CFUs) (Nagachandrabose et al., 2022).

The percentage of egg mass parasitization by the fungal biocontrol agents *P. lilacinum* and *T. asperellum* was also assessed (Nagachandrabose et al., 2022). For this, 10 egg masses were manually collected from each treatment root subsample using forceps, surface-sterilized with 1% sodium hypochlorite (NaOCl), rinsed twice with distilled water, and placed on PDA and YMA media in 90-mm Petri plates. The plates were incubated at 28 ± 3°C for 15 days, after which the fungi colonizing the eggs were identified based on their distinctive morphological characteristics. The percentage parasitization was calculated using the formula:


Percentage parasitization=(Number of infected egg masses/Total egg masses)×100.

### 2.10 Growth metrics and yield assessment

Plant growth parameters, such as the number of branches per plant, the number of leaves per plant, plant height (cm), and fruit yield (kg/plant), were measured from five randomly selected and tagged plants. Measurements were taken at 15-day intervals throughout the crop cycle, and mean values were calculated. Fruit yield per replicate plot was recorded at each harvest, and the cumulative yield was calculated to determine the total fruit yield in tons per hectare (ha).

### 2.11 Statistical analysis

The normality of the data was assessed using the Shapiro-Wilk test. Nematode population data were log-transformed to ensure homogeneity before analysis. A one-way ANOVA was performed, and treatment means were compared using Tukey’s range test. The data analysis was done using SPSS 16.0 for Windows software (SPSS Inc., Chicago, IL, USA). The results are presented using untransformed data.

## 3 Results

### 3.1 Efficacy of eggplant-tomato grafting and bio-inputs on *M. incognita*

At the start of the experiment, the initial population of *M. incognita* second-stage juveniles (J2) did not show any significant variation among the treatment plots at either location (**Tables 1**, **2**). Nevertheless, a comparatively higher pre-treatment infestation was recorded at Location I, averaging 435.1 ± 15.2 J2 per 200 cm³ of soil, whereas Location II recorded a slightly lower mean of 381.5 ± 16.4 J2 per 200 cm³. As the trials progressed, significant differences in the J2 populations at harvest were observed across the six treatment groups in both locations (Location I: F = 7.6; df = 5, 15; P< 0.001; Location II: F = 6.7; df = 5, 15; P< 0.001). By the end of the season, final J2 population densities varied between 146.9 and 604.7 J2 per 200 cm³ in Location I, and between 135.5 and 586.3 J2 per 200 cm³ in Location II, indicating differential treatment effects on nematode suppression.

**Table 1:** Impact of eggplant-tomato grafting, alone and with bio-inputs, on *Meloidogyne incognita* infestation in tomato – Location I (Vandikaranur).

Treatments	Population of *Meloidogyne incognita* J2 in 200 cm^3^ soil		Root gall index	Number of females/g root	Number of egg mass/g root	Number of eggs/egg mass
	At planting	At harvest				
EG 203-Tomato graft + Bio-inputs	432.7 ± 12.6 a	**146.9 ± 8.2 e**	**2.1 e**	**12.3 ± 1.2 e**	**7.4 ± 0.7 e**	**177.3 ± 8.3 e**
TS 03-Tomato graft + Bio-inputs	425.5 ± 15.1 a	339.0 ± 12.7 c	3.3 c	26.7 ± 2.3 c	24.2 ± 1.9 c	256.8 ± 12.6 c
Tomato + Bio-inputs	434.8 ± 11.2 a	351.5 ± 18.4 c	3.6 c	27.2 ± 2.7 c	25.8 ± 2.1 c	268.8 ± 13.1 c
EG 203- Tomato graft	441.4 ± 18.3 a	269.0 ± 14.8 d	2.7 d	20.1 ± 2.2 d	12.3 ± 0.7 d	214.5 ± 10.6 d
RS-TS 03-Tomato graft	436.7 ± 14.8 a	470.6 ± 19.1 b	4.0 b	30.9 ± 3.6 b	27.5 ± 1.7 b	243.1 ± 11.8 b
Tomato	439.6 ± 19.2 a	604.7 ± 38.4 a	4.7 a	36.3 ± 3.8 a	32.6± 2.3 a	286.7 ± 13.3 a

According to Tukey's range test, means (±SEM) values in a column followed by the same alphabet (s) are not significantly different at p = 0.05.

Bold values indicate significantly superior effects compared to other treatments (p < 0.05).

**Table 2:** Impact of eggplant-tomato grafting, alone and with bio-inputs, on *Meloidogyne incognita* infestation in tomato – Location II (Karadimadai).

Treatments	Population of *Meloidogyne incognita* J2 in 200 cm^3^ soil		Root gall index	Number of females/g root	Number of egg mass/g root	Number of eggs/egg mass
	At planting	At harvest				
EG 203-Tomato graft + Bio-inputs	387± 15.8 a	**135.5 ± 10.1 e**	**2.0 e**	**10.5 ± 0.8 e**	**6.5 ± 0.6 e**	**142.8 ± 8.2 d**
RS-TS 03-Tomato graft + Bio-inputs	371± 17.1 a	309.7 ± 16.4 c	3.3 c	20.4 ± 1.3 c	19.3 ± 0.9 cb	214.4 ± 9.6 b
Tomato + Bio-inputs	395 ± 16.1 a	312.1 ± 18.2 c	3.5 c	21.6 ± 1.8 c	20.3 ± 1.3 b	216.1 ± 12.4 b
EG 203- Tomato graft	378 ± 15.8 a	223.0 ± 13.6 d	2.6 d	15.5 ± 0.9 d	9.3 ± 0.8 d	180.9 ± 10.7 c
RS-TS 03-Tomato graft	382± 17.3 a	420.9 ± 19.6 b	4.0 b	24.1 ± 1.7 b	21.7 ± 1.7 b	219.4 ± 11.1 b
Tomato	376± 16.4 a	586.3± 25.3 a	4.5 a	27.7 ± 2.1 a	24.7 ± 1.8 a	238.3 ± 13.6 a

According to Tukey's range test, means (±SEM) values in a column followed by the same alphabet (s) are not significantly different at p = 0.05.

Bold values indicate significantly superior effects compared to other treatments (p < 0.05).

In both experimental locations, the *M. incognita* populations in soil were significantly reduced in the EG 203-tomato grafts treated with bio-inputs, which proved to be the most effective treatment. Specifically, this combination led to a reduction of 77.7% in Location I and 76.8% in Location II when compared to the untreated tomato Shivam^®^ control. Following this, the EG 203-tomato grafts without bio-inputs ranked second in effectiveness, achieving 55.5% and 61.9% reductions at Locations I and II, respectively. Meanwhile, intermediate suppression levels were observed in the TS 03-tomato graft with bio-inputs (43.9–47.1%) and tomato Shivam^®^ with bio-inputs (41.8–46.7%). By contrast, the TS 03-tomato grafts and tomato Shivam^®^ without bio-input treatments exhibited the highest *M. incognita* population densities, confirming their relatively poor nematode suppression performance.

**Figure 3:**
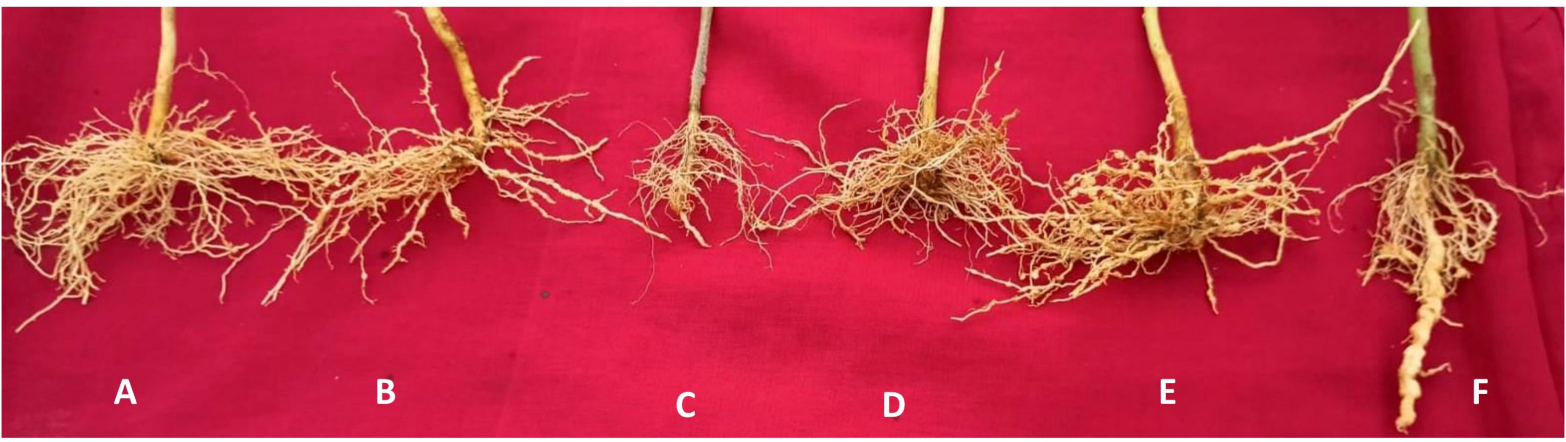
**(A)** Roots of EG 203-tomato Shivam® graft with bio-inputs with fewer galls, **(B)** TS 03-tomato Shivam® graft with bio-inputs with moderate galls, **(C)** tomato Shivam® with bio-inputs with moderate galls, **(D)** EG 203-tomato Shivam® graft without bio-inputs with moderate galls, **(E)** TS 03 -tomato Shivam® graft without bio-inputs with higher galls, and **(F)** tomato Shivam® alone (non-treated control) with heavy galls. **Figure 4**. Left: More gravid females of M. incognita in roots of tomato Shivam® alone (non-treated control), Right: Fewer gravid females of M. incognita in roots of EG 203-tomato Shivam® graft with bio-inputs.

The lowest root gall index (2.0–2.1) was consistently recorded in EG 203-tomato graft plants treated with bio-inputs across both locations (**Figure 3**). This treatment demonstrated superior nematode suppression. It was closely followed by EG 203-tomato grafts without bio-inputs, which recorded slightly higher root gall indices of 2.6 and 2.7. In contrast, moderate galling was observed in TS 03-tomato grafts and tomato Shivam^®^ plants treated with bio-inputs, with indices ranging between 3.3 and 3.6. Notably, the highest levels of gall formation were found in untreated TS 03-tomato graft and tomato Shivam^®^ plants, with root gall indices ranging from 4.0 to 4.7 (**Tables 1**, **2**). These findings clearly illustrate the effectiveness of both grafting and bio-input application in minimizing root gall severity.

**Figure 4:**
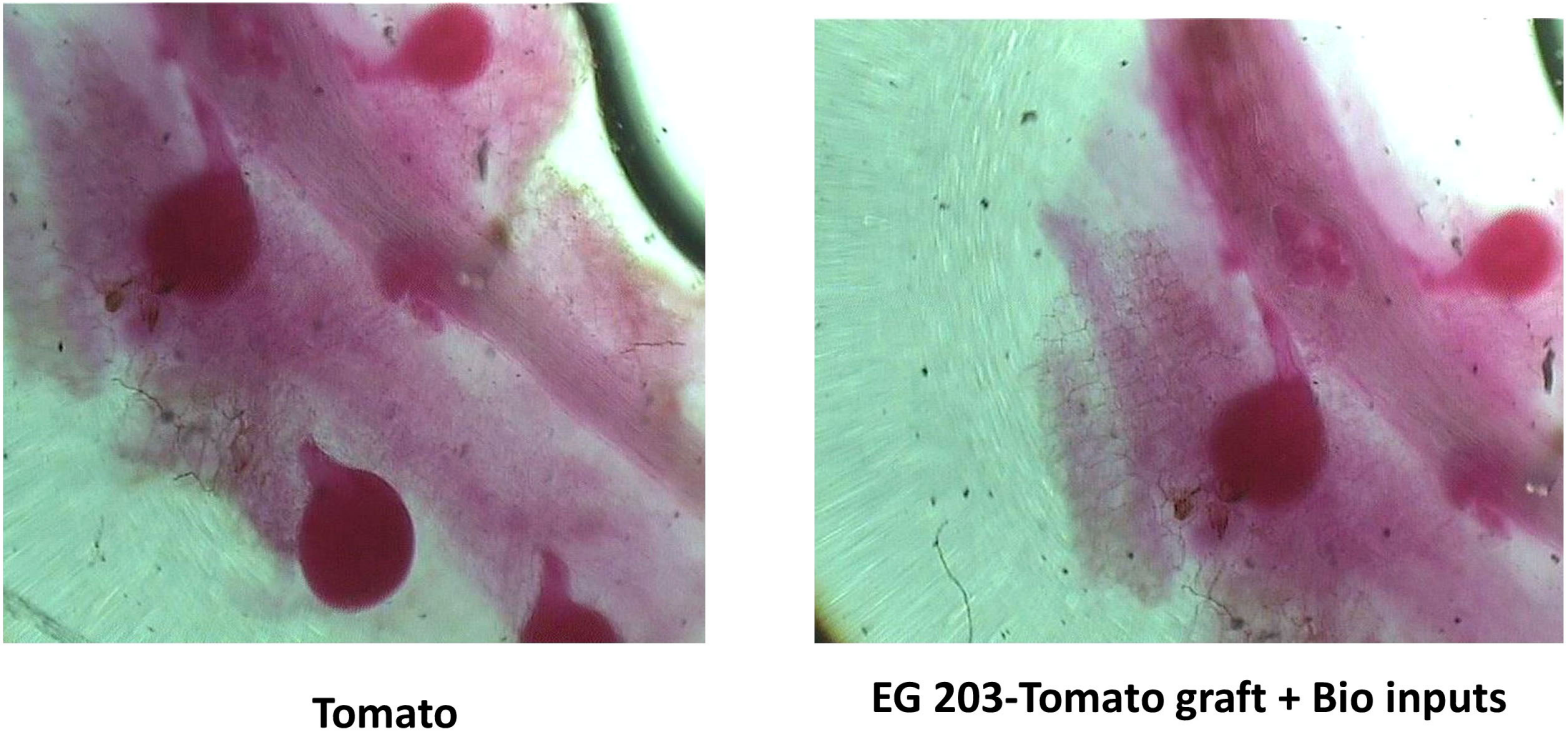
Left: More gravid females of *M. incognita* in roots of tomato Shivam^®^ alone (non-treated control), Right: Less number of gravid females of *M. incognita* in roots of EG 203-tomato Shivam^®^ graft with bio-inputs.

The adult female population of *M. incognita* was significantly lower (P< 0.05) in EG 203-tomato grafts treated with bio-inputs, with only 12.3 females per g of root recorded in Location I and 10.5 in Location II (**Figure 4**). In sharp contrast, untreated tomato Shivam^®^ plants harbored the highest female populations, registering 36.3 females/g root in Location I and 27.7 in Location II. Meanwhile, EG 203-tomato grafts without bio-inputs exhibited moderately reduced infection rates, with 15.5 to 20.1 females/g root. Similarly, TS 03-tomato grafts and tomato Shivam^®^ treated with bio-inputs showed intermediate female populations, ranging from 20.4 to 26.7 and 21.6 to 27.2 females/g root, respectively (**Tables 1** and **2**). These results reinforce the effectiveness of the EG 203-tomato graft and bio-input combination in significantly limiting nematode reproduction in root tissues.

The number of egg masses per g of root varied significantly across treatments in both locations (Location I: F = 8.1; df = 5, 15; P< 0.001; Location II: F = 7.3; df = 5, 15; P< 0.001), with counts ranging from 7.4 to 32.6 in Location I and 6.5 to 24.7 in Location II. Among the treatments, untreated tomato Shivam^®^ and TS 03-tomato grafts recorded the highest egg mass production, indicating greater nematode reproduction in the absence of bio-inputs. Furthermore, the egg count per egg mass also showed statistically significant variation across treatments (Location I: F = 5.6; df = 5, 15; P< 0.001; Location II: F = 6.3; df = 5, 15; P< 0.001). Notably, EG 203-tomato grafts treated with bio-inputs exhibited the lowest egg counts per mass, reflecting a 38.1–40.0% reduction compared to untreated tomato Shivam^®^ plants (**Tables 1**, **2**). These findings demonstrate the combined efficacy of resistant rootstock and microbial inputs in suppressing nematode fecundity.

### 3.2 Efficacy of eggplant-tomato grafting and bio-inputs on crop biometry

**Figure 5:**
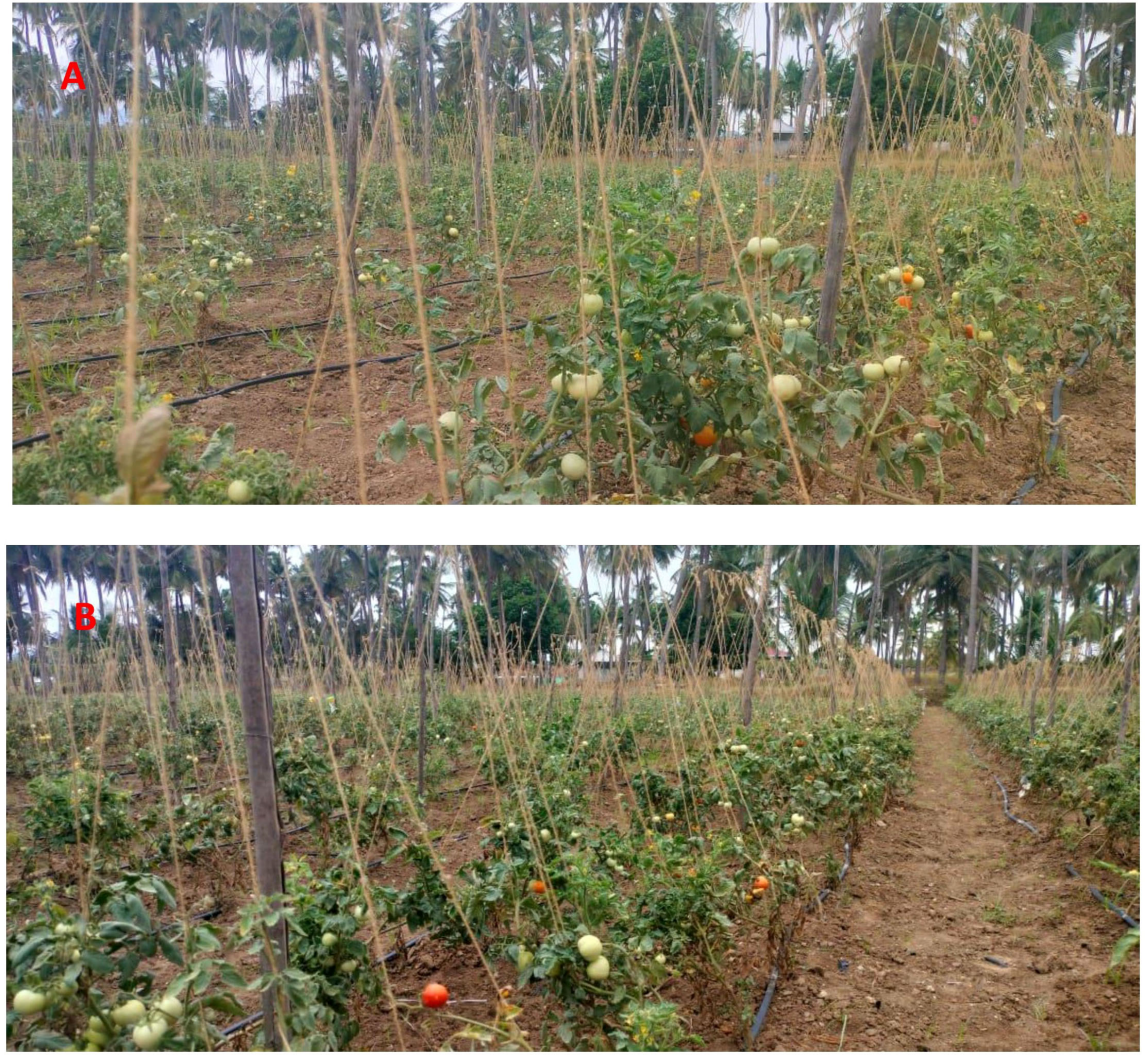
**(A)** Field view of EG 203-tomato Shivam^®^ graft with bio-inputs plots showing plants with good biometric characteristics. **(B)** Field view of tomato Shivam^®^ alone (non-treated control) with patchy appearance and less biometric characteristics.

Growth and yield parameters, including the number of branches per plant, number of leaves per plant, plant height, fruit yield per plant, and total fruit yield, were significantly enhanced in EG 203-tomato grafts treated with bio-inputs compared to other treatments (**Tables 3**, **4**; **Figure 5**). Moreover, statistical analysis revealed significant differences in all measured biometric parameters between EG 203-tomato and TS 03-tomato grafts. Specifically, the number of branches (F = 84.85; df = 5,15; P< 0.001), number of leaves (F = 532.01; df = 5,15; P< 0.001), plant height (F = 564.45; df = 5,15; P< 0.001), and number of fruits (F = 1038.34; df = 5,15; P< 0.001) showed highly significant variation among the treatments. These results indicate that combining the resistant EG 203 rootstock with beneficial microbial consortia positively influenced vegetative growth and fruit production under nematode-infested conditions.

**Table 3:** Effect of eggplant-tomato grafting, with and without bio-inputs, on growth and yield metrics of tomato – Location I (Vandikaranur).

Treatments	Number of branches/plants	Number of leaves/plants	Plant height (cm)	Fruit yield (kg/plant)	Fruit yield (t/ha)
EG 203-Tomato graft + Bio-inputs	**3.7 ± 0.3 a**	**84.3 ± 3.1 a**	**54.6± 1.8 a**	**10.8 ± 0.7 a**	**15.4 ± 0.8 a**
RS-TS 03-Tomato graft + Bio-inputs	2.6 ± 0.2 c	56.4 ± 2.3 d	43.8 ± 1.5 c	5.1 ± 0.3 d	12.7 ± 0.6 bc
Tomato + Bio-inputs	3.3 ± 0.3 b	75.2 ± 3.4 b	52.5± 1.7 b	8.8 ± 0.7 b	14.0 ± 0.7 ab
EG 203- Tomato graft	2.2 ± 0.2 d	50.5 ± 2.2 e	43.6 ± 1.6 d	4.2 ± 0.4 e	11.4 ± 0.6 cd
RS-TS 03-Tomato graft	1.9 ± 0.1 d	47.2± 1.8 f	36.8 ± 1.4 e	3.9 ± 0.3 f	11.1 ± 0.6 d
Tomato	3.2 ± 0.2 b	68.3 ± 2.4 c	53.7± 1.9 b	7.8 ± 0.6 c	13.3 ± 0.7 bc

According to Tukey's range test, means (±SEM) values in a column followed by the same alphabet (s) are not significantly different at p = 0.05.

Bold values indicate significantly superior effects compared to other treatments (p < 0.05).

**Table 4:** Effect of eggplant-tomato grafting, with and without bio-inputs, on growth and yield metrics of tomato – Location II (Karadimadai).

Treatments	Number of branches/plant	Number of leaves/plant	Plant height (cm)	Fruit yield (kg/plant)	Fruit yield (t/ha)
EG 203-Tomato graft + Bio-inputs	**3.1 ± 0.2 a**	**81 .3 ± 2.4 a**	**54.7± 1.9 a**	**11.5 ± 0.6 a**	**13.6 ± 0.7 a**
RS-TS 03-Tomato graft + Bio-inputs	2.3 ± 0.1 d	59.2± 1.8 d	42.3.8 ± 1.4 d	6.9 ± 0.3 d	11.1 ± 0.6 b
Tomato + Bio-inputs	2.8 ± 0.2 b	71.5 ± 2.2 b	51.6 ± 1.6 b	9.4 ± 0.4 b	13.2 ± 0.7 a
EG 203- Tomato graft	1.7 ± 0.3 e	54.2 ± 3.4 e	41.5± 1.7 e	5.8 ± 0.7 e	9.8 ± 0.8 c
RS-TS 03-Tomato graft	1.6 ± 0.2 f	49.4 ± 2.3 f	36.8.8 ± 1.5 f	4.6± 0.3 f	8.7 ± 0.6 c
Tomato	2.7 ± 0.3 c	64.3 ± 3.1 c	51.6± 1.8 c	8.3 ± 0.7 c	12.9 ± 0.8 ab

According to Tukey's range test, means (±SEM) values in a column followed by the same alphabet (s) are not significantly different at p = 0.05.

Bold values indicate significantly superior effects compared to other treatments (p < 0.05).

Although EG 203-tomato grafts showed a slight delay in establishment during the first week after planting, they recovered by the second week and exhibited growth comparable to conventional tomato plants. Moreover, the grafted plants remained productive in the field for an additional month beyond the traditional tomatoes, enabling extended harvests and increased yield potential.

At both locations, EG 203-tomato grafts treated with bio-inputs exhibited superior growth and yield performance. These plants reached greater heights (54.6–54.7 cm), developed more branches (3.1–3.7), and produced a higher number of leaves (81.3–84.3), resulting in a fruit yield of 10.8–11.5 kg per plant. In contrast, TS 03-tomato grafts without bio-inputs showed significantly reduced growth, with shorter plant heights (36.8 cm), fewer branches (1.6–1.9), and leaves (47.2–49.4), culminating in a markedly lower fruit yield of 3.9–4.6 kg per plant. Notably, the application of bio-inputs consistently improved growth and yield parameters, regardless of the graft type, highlighting their role in enhancing tomato productivity.

### 3.3 Survival and parasitization potential of introduced bio-agents

**Figure 6:**
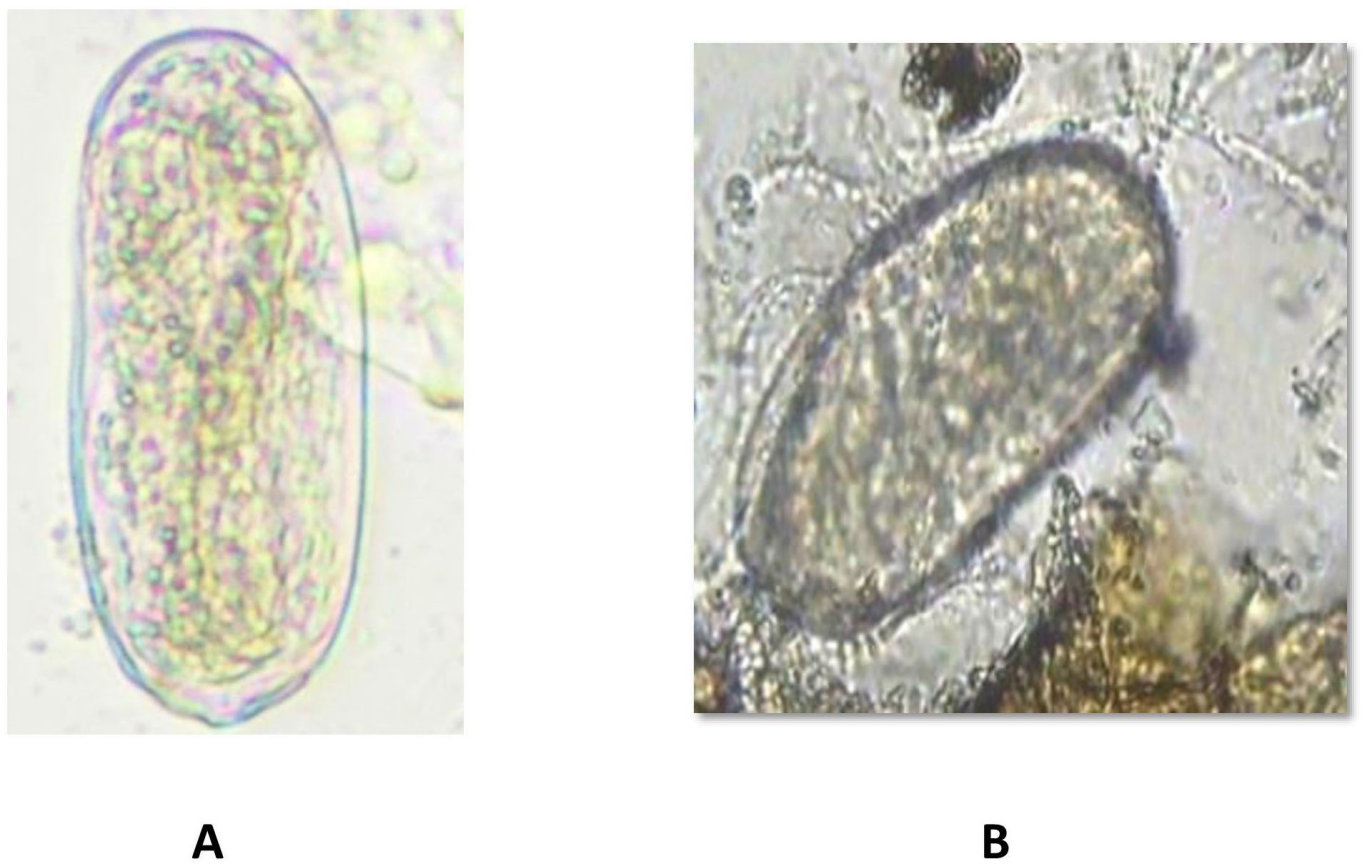
**(A)** *Meloidogyne incognita* egg in tomato Shivam^®^ alone (non-treated control) remaining healthy. **(B)** *Meloidogyne incognita* egg in EG 203-tomato Shivam^®^ graft with bio-inputs plots with *Purpureocilium lilacinum* parasitization.

**Figure 7:**
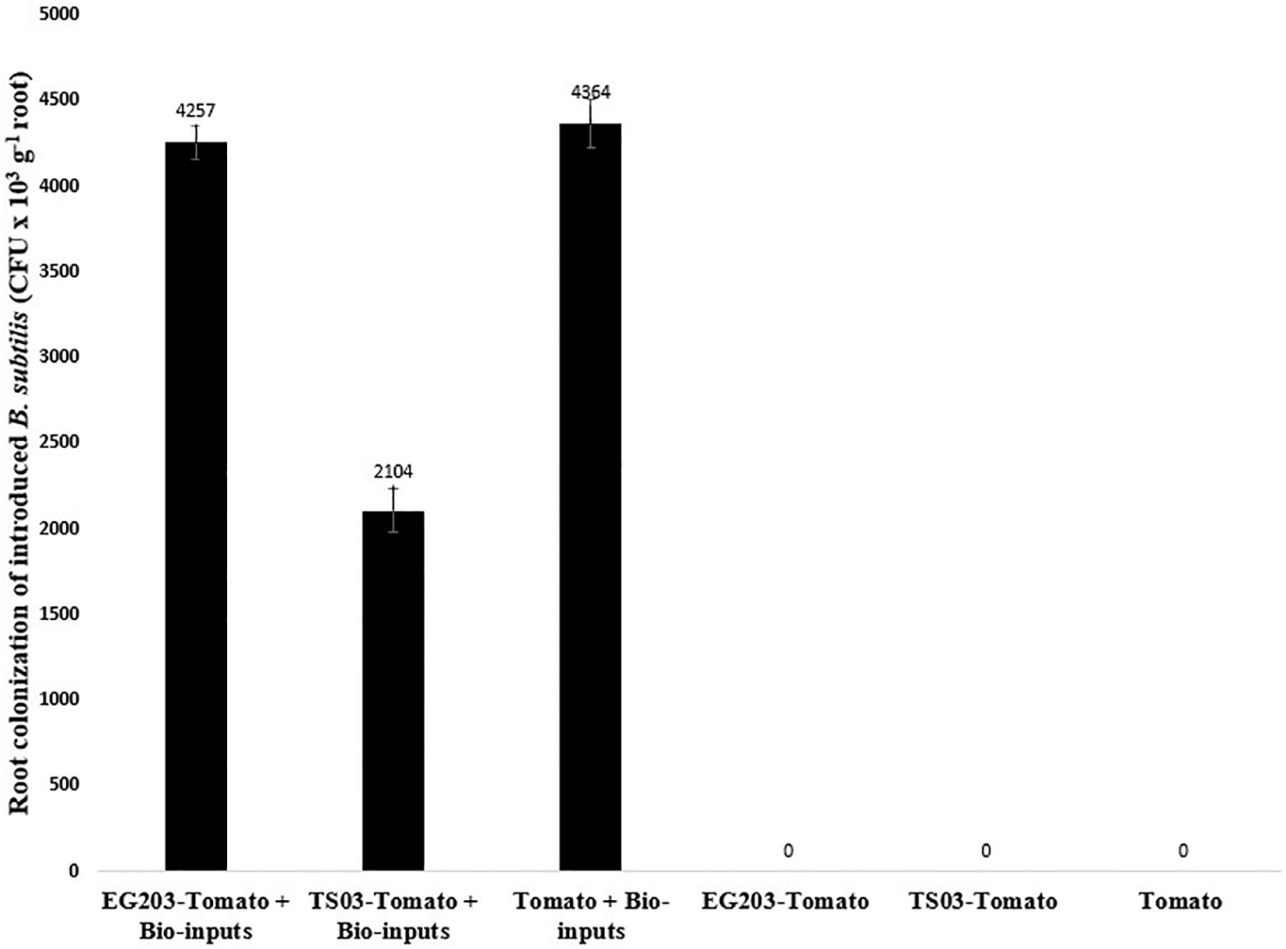
The colonization of grafted and non-grafted tomato roots by the introduced *Bacillus subtilis*.

**Figure 8:**
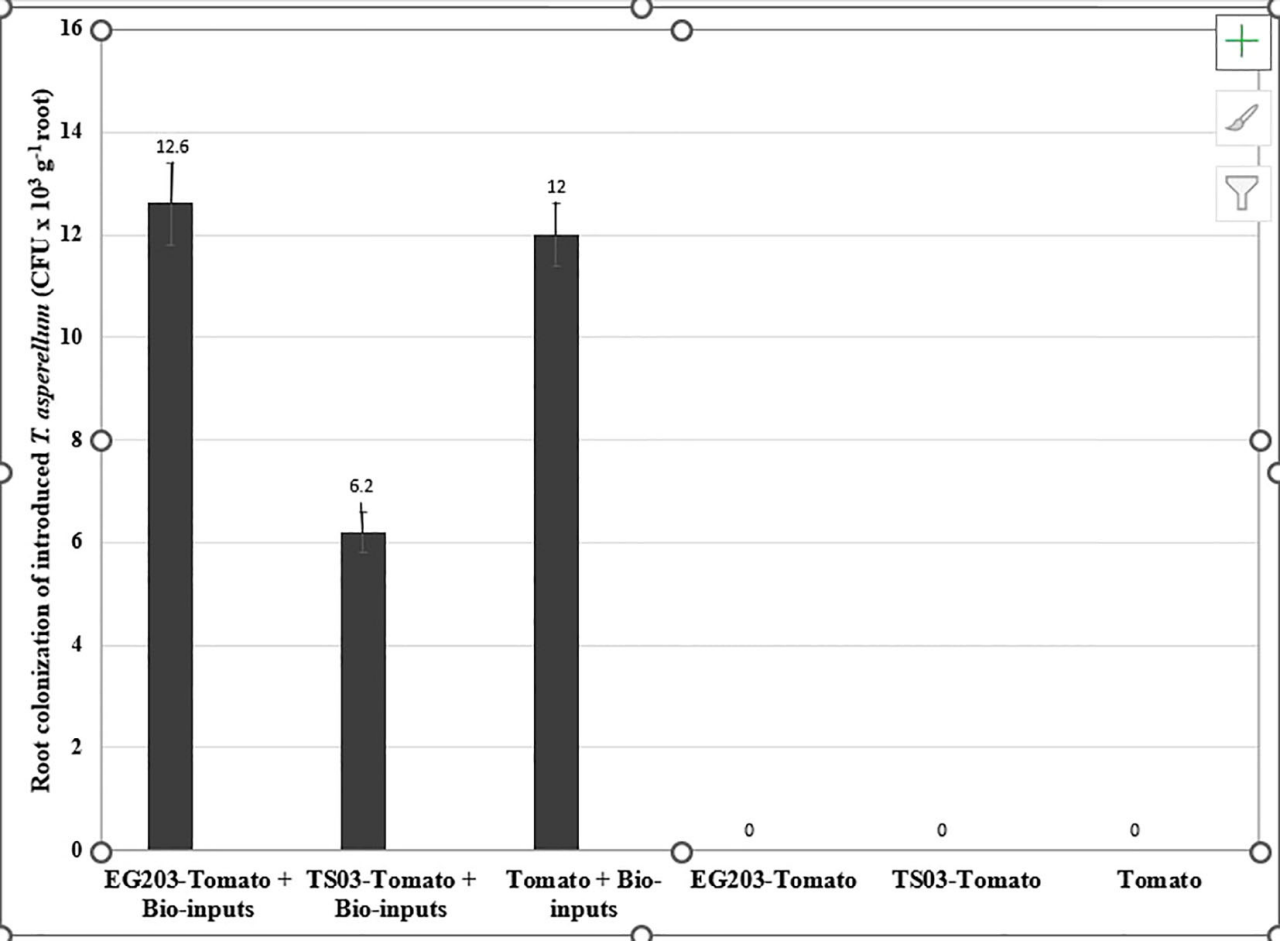
The colonization of grafted and non-grafted tomato roots by the introduced *Trichoderma asperellum*.

**Figure 9:**
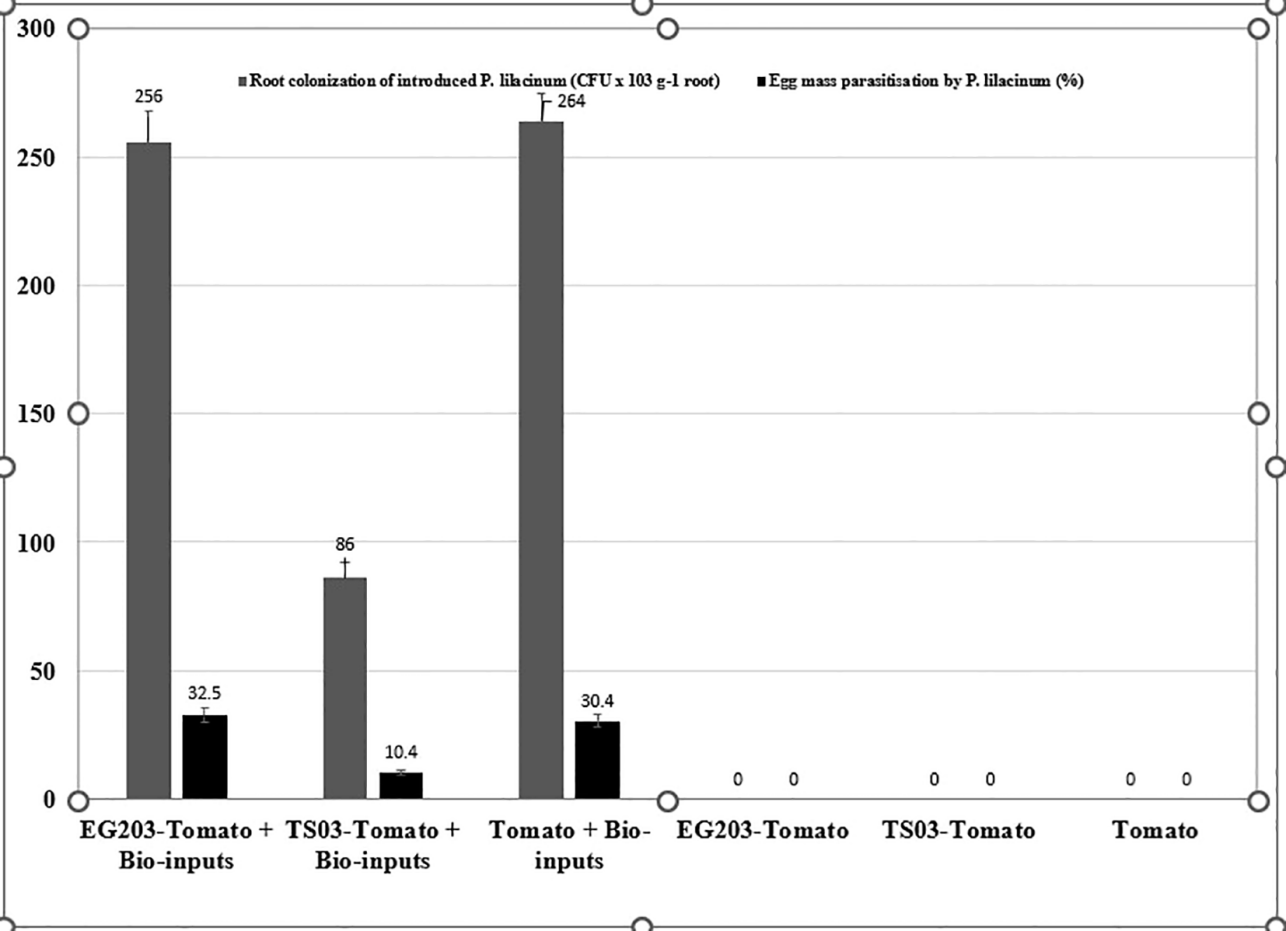
The colonization of grafted and non-grafted tomato roots was caused by the introduction of *Purpureocillium lilacinum* and its parasitization percentage on *M. incognita* egg mass.

The introduced bio-agents were successfully re-isolated from tomato roots, confirming their establishment and parasitic activity on *M. incognita* egg masses located on root surfaces (**Figure 6**). However, colonization potential varied significantly among treatments. Notably, colonization was markedly lower in TS 03-tomato grafts compared to both EG 203-tomato grafts and the non-grafted tomato Shivam^®^. In the EG 203-tomato grafts and tomato Shivam^®^, colonization levels of *B. subtilis* and *T. asperellum* were approximately twice as high (**Figures 7**, **8**), while *P. lilacinum* exhibited a three-fold increase in colonization relative to the TS 03-tomato grafts (**Figure 9**). Similarly, the parasitization of egg masses by *P. lilacinum* was three times lower in TS 03 grafts (**Figure 9**). These observations collectively underscore the superior survival, colonization, and biocontrol efficacy of the bio-agents in EG 203-tomato grafts and tomato Shivam^®^, highlighting the importance of compatible graft combinations in enhancing biological control outcomes.

## 4 Discussion

Grafting tomato scions with desirable horticultural traits onto rootstocks resistant to *Meloidogyne incognita* offers a viable management strategy for resource-limited tomato farmers. Previous studies have demonstrated the efficacy of root-knot nematode-resistant tomato varieties, such as ‘Celebrity,’ ‘Big Beef,’ and ‘Jetsetter’ as rootstocks for nematode-susceptible commercial cultivars like ‘Tropimech’ and ‘Power’ (Owusu et al., 2016). Similarly, grafting bacterial wilt-resistant varieties has effectively managed *Ralstonia solanacearum* and *M. incognita* using the same rootstocks (Kunwar et al., 2015). Recent advancements have expanded resistance strategies to include solanaceous plants for grafting, leveraging their strong resistance to *M. incognita* and facilitating the transfer of Mi gene resistance from *Solanum torvum* to cultivated tomato (Fullana et al., 2024).

The resistance conferred by such rootstocks is primarily attributed to the presence of Mi genes, which trigger a hypersensitive response (HR) in infected root tissues, leading to localized cell death and halting nematode development. These genes inhibit nematode penetration, feeding site formation, and reproduction. In addition, resistant rootstocks often possess enhanced lignification, thicker epidermal layers, and elevated levels of defensive enzymes and secondary metabolites, such as peroxidase, phenolics, and reactive oxygen species, which further fortify the root tissues against pathogen invasion. Collectively, these factors prevent nematode establishment and damage, making the rootstock an effective barrier against *M. incognita*.

Our study presents the first report of *M. incognita* resistance in an eggplant-tomato graft, specifically ‘EG 203-tomato,’ created by grafting a *R. solanacearum*-resistant eggplant rootstock (EG 203) with the commercial tomato cultivar Shivam^®^. Field evaluations of two eggplant rootstocks, EG 203 and TS 03, both resistant to bacterial wilt, demonstrated their potential to confer resistance to *M. incognita*. EG 203-tomato grafts exhibited the lowest root gall index (2.6–2.7) across the experimental locations, Vandikaranur and Karadimadai villages, Coimbatore, Tamil Nadu, India. Conversely, TS 03-tomato grafts showed a higher gall index (4.0). These findings suggest that EG 203-tomato grafts resistant response, while TS 03-tomato grafts show susceptibility based on tomato resistance rating (Taylor and Sasser, 1978).

The resistance of EG 203-tomato grafts was evident in their ability to suppress *M. incognita* populations, even without bio-inputs. Reductions of 55.5–61.9% in soil M. incognita J2 populations, 45% in female nematode counts in roots, 62% in egg masses, and 24–25% in eggs per mass were observed. These results indicate that EG 203-tomato grafts may deploy both pre-infectional and post-infectional resistance mechanisms, disrupting nematode penetration, reproduction, and life cycle progression. Pre-infectional mechanisms may involve structural barriers such as thicker root epidermis and increased lignification, which hinder nematode entry. Post-infectional defenses may include localized hypersensitive responses (HR), oxidative bursts, and the upregulation of defense-related enzymes such as peroxidases, chitinases, and phenylalanine ammonia-lyase, which inhibit nematode feeding and development. These responses contribute to the suppression of giant cell formation and limit nematode reproduction within the root tissue. Additionally, resistance may be mediated by the expression of Mi-gene homologs, which are known to confer effective resistance against *Meloidogyne* spp. in certain solanaceous rootstocks (Abad et al., 2003; Williamson and Kumar, 2006). Further studies under controlled conditions are needed to confirm these resistance mechanisms.

The application of bio-inputs, including neem oil cake in soil and seedling drenches in the nursery and microbial agents (*B. subtilis*, *T. asperellum*, and *P. lilacinum*) in the field, further enhanced the suppression of the nematode *M. incognita*. Neem cake has been previously shown to reduce nematode population through its nematicidal component, azadirachtin, which inhibits *M. incognita* J2 mobility, root penetration, and reproduction while also activating systemic resistance, by inducing defense enzymes such as peroxidase, polyphenol oxidase, phenylalanine ammonia lyase, and superoxide dismutase, in bio-input-treated plants (Chandrawat et al., 2020; Gautam et al., 2021; d’Errico et al., 2023; Ambrogioni et al., 2003).

Among the microbial bioagents, *viz*., *B. subtilis*, *T. asperellum*, and *P. lilacinum*, *B. subtilis* exhibited higher colonization efficiency in tomato roots, confirming its active role in reducing *M. incognita* populations when applied through the soil, seed treatment, root dip, and seedling drench. In this study, *B. subtilis* was successfully re-isolated from tomato roots following its application in the nursery and at planting, confirming its active role in reducing *M. incognita* populations under field conditions. Previous studies have demonstrated the nematode suppressive activity of *B. subtilis* through the production of acetic acids, proteases, hydrogen cyanide, and indole acetic acid, as well as its ability to induce systemic resistance (Siddiqui, 2002; Singh and Siddiqui, 2010; Eltayeb, 2017; Basyony and Abo-Zaid, 2018).

Similarly, *T. asperellum* was re-isolated from tomato roots, although it did not parasitize *M. incognita* eggs in this study, suggesting that the strain (Tv 1) lacked egg-parasitic activity. Instead, its efficacy likely stemmed from its ability to induce systemic resistance by activating defense enzymes such as peroxidase, chitinase, and phenylalanine ammonia lyase (Naserinasab et al., 2011; Singh et al., 2017).

*P. lilacinum* was also effectively re-isolated and demonstrated significant parasitism of *M. incognita* eggs, aligning with previous reports of its nematode-suppressive potential (Das and Waquar, 2021). Its ability to produce toxic metabolites, including eucinostatins and paecilotoxins, and disrupt giant cell formation likely contributed to the observed reduction in nematode populations (Nagachandrabose et al., 2022).

Beyond nematode suppression, bio-input treatments consistently enhanced growth and yield metrics in EG 203-tomato grafts. Grafted tomatoes typically face yield trade-offs when using pathogen-resistant rootstocks (Owusu et al., 2016). However, our findings challenge this paradigm, as integrating bio-inputs with EG 203-rootstock improved resistance to biotic stresses and enhanced plant growth and productivity. This yield improvement is likely attributed to a combination of factors: the suppressive effects of the EG 203 rootstock, bio-input induced nematode suppression, and the growth-promoting properties of neem cake (Rizvi et al., 2015), *B. subtilis* (de O. Nunes et al., 2023), and *P. lilacinum* (Baron et al., 2020).

Overall, this study highlights the potential of EG 203-tomato grafts as an efficient and sustainable solution for managing *M. incognita* in tomato cultivation. Integrating grafting with bio-input treatments offers a dual benefit of resistance to nematode infections and enhanced crop productivity, positioning this approach as a promising strategy for sustainable tomato production in resource-limited farming systems.

## 5 Conclusion

This study demonstrates that grafting tomato onto the bacterial wilt-resistant rootstock EG 203 significantly enhances resistance to *M. incognita* under field conditions. When combined with selected biological agents *B. subtilis*, *T. asperellum*, and *P. lilacinum*—applied during nursery and field stages, this integrated strategy effectively suppresses nematode populations, reduces root damage, and improves plant growth and fruit yield. The EG 203 rootstock not only delays senescence but also sustains productivity, offering both agronomic and economic benefits to farmers. Scientifically, this work contributes to the growing evidence supporting rootstock-mediated resistance and highlights the synergistic role of biological control in nematode management. These findings present a promising, eco-friendly alternative to chemical nematicides and support the adoption of sustainable practices in tomato cultivation. Further research to dissect the underlying resistance mechanisms will enhance our understanding and scalability of this grafting–bio-input integration approach.

## Data Availability

The raw data supporting the conclusions of this article will be made available by the authors, without undue reservation.
